# Co-activator p120 is increased by gonadotropins in the rat ovary and enhances progesterone receptor activity

**DOI:** 10.1186/1477-7827-4-50

**Published:** 2006-10-03

**Authors:** Miki Yoshino, Tetsuya Mizutani, Kazuya Yamada, Takashi Yazawa, Hiroko Ogata-Kawata, Toshio Sekiguchi, Takashi Kajitani, Kaoru Miyamoto

**Affiliations:** 1 Department of Biochemistry, Faculty of Medical Sciences, University of Fukui, Shimoaizuki, Matsuoka, Fukui 910-1193, Japan; 2 CREST, JST (Japan Science and Technology), Japan

## Abstract

**Background:**

Ovarian follicular development is primarily dependent on pituitary gonadotropins. Identification of gonadotropin-inducible genes in the ovary is one of the effective approaches for the study of follicular development. In this study we identify rat homologue of p120, a nuclear transcription co-activator, as one of the FSH inducible genes in the rat granulosa cells.

**Methods:**

A full-length cDNA encoding rat p120 was cloned, and expression of the gene in the ovary was examined by Northern blotting. Tissue localization of p120 was examined by in situ hybridization. Cellular functions of p120 were studied by co-transfection of rat p120 gene together with estrogen receptor (ER)-alpha, ER-beta, androgen receptor (AR), or progesterone receptor (PR) genes.

**Results:**

A full-length cDNA encoding rat p120 was characterized as a protein with 957 amino acid residues. Rat p120 was expressed ubiquitously, but strongly in the ovary and the testis. Expression of p120 mRNA was also induced in vivo by PMSG or PMSG/hCG treatment. Strong expression of p120 mRNA was observed in the granulosa cells of pre-ovulatory large antral follicles. Progesterone receptor was co-localized with p120 in the large antral follicles. Co-transfection experiments revealed that rat p120 activated AR, ER-alpha, ER-beta, and PR in the presence of their respective ligands.

**Conclusion:**

These observations suggest that rat p120 is strongly induced in the ovarian granulosa cells, and may work together with PR in the granulosa cells of ovulatory follicles to promote the ovulation process.

## Background

Follicular growth is primarily controlled by pituitary gonadotropins LH and FSH in assistance with various intraovarian factors that also play essential and significant roles in development. FSH stimulates proliferation and differentiation of granulosa cells of small follicles in the ovary, and promotes their development to pre-ovulatory follicles [1-3]. During the differentiation of the granulosa cells, steroid hormone synthesizing enzymes are induced in the cells [4]. Estrogen and progesterone produced in the ovary work primarily on the uterus or the mammary gland, but the steroids also regulate pituitary gonadotropin secretion through positive and negative feedback systems of hypothalamus-pituitary-ganadal axis [5]. These steroid hormones also work on the ovary in either a paracrine or an autocrine fashion [6]. Progesterone is known to be involved in the ovulation process [7,8], and estrogen receptor knockout results in the malfunction of the mouse ovary [9]. Deficiencies of progesterone receptors or estrogen receptors result in various defects of reproductive functions, such as abnormal follicular development, anovulation, and infertility [10-12]. Both estrogen and progesterone receptors belong to the nuclear receptor super-family proteins that activate the transcription of their target genes when the steroid hormones or ligands bind to them [12,13]. The nuclear hormone receptors generally bind to the respective hormone responsive elements (HREs) within the upstream regions of the target genes, and activate their transcription [14-16]. Co-activators are required to form active protein complexes with the hormone nuclear receptors and the basic transcription machinery proteins for the target gene transcription [17,18]. Co-activators p160/SRC and CBP/p300 are known to interact with the nuclear hormone receptors at the C-terminal AF-2 region through the LXXLL-motif (NR box) [19-21]. Knockout mice of steroid receptor co-activator 3 (SRC-3), a member of p160 family, exhibit infertility [22], and those of nuclear receptor interacting protein 1 (Nrip1/RIP140) are also infertile due to the blockage of the oocyte release from the Graafian follicles by the ovulatory stimuli [23]. These reports indicate that co-activators are also important for follicular development and ovulation. Recently we identified many gonadotropin inducible genes expressed in the ovary by subtraction cloning and DNA microarray analyses, and found that p120, a member of nuclear co-activators, was strongly induced in the rat cultured granulosa cells by FSH. The co-activator p120 was originally isolated as a novel nuclear co-activator for thyroid hormone receptor [24]. Further studies revealed that p120 also interacts with 9-cis-retinoic acid receptor (RXR) functioning as a co-activator of these nuclear receptors [25]. In this study, we examined expression and localization of p120 gene in the ovary during the follicular development. In addition, we examined functions of rat p120 as a nuclear co-activator by transfection experiments, and showed that p120 may promote the function of the progesterone receptors in the ovary during the ovulation.

## Materials and methods

### Materials

The Oligotex dT-30 super, T7- and SP6-RNA polymerases were obtained from Roche Molecular Biochemicals (Mannheim, Germany). The TRIZOL reagent, an RNase inhibitor, a Superscript II reverse transcriptase, an expression vector pcDNA3, and a Lipofectamine PLUS reagent were obtained from Invitrogen (Groningen, Netherlands). The dual luciferase reporter assay system, a pGEM-T Easy vector, and pGL3-Basic and pRL-CMV vectors were purchased from Promega (Madison, WI). A PCR-selected cDNA subtraction kit and ExpressHyb, a hybridization solution, were purchased from Clontech (Palo Alto, CA). The QIAGEN plasmid kit was purchased from QIAGEN (Hilden, Germany). The TaKaRa *Bca *BEST Labeling Kit was purchased from TaKaRa Shuzo (Kyoto, Japan). The [ganma-^32^P] deoxycytidine triphosphate (dCTP; 111 TBq/mmol) and [^35^S] cytidine triphosphate (CTP; 46.2 TBq/mmol) were obtained from NEN Life Science Products (Wilmington, DE). The Fluorolinks Cy3-dCTP and Cy5-dCTP were purchased from Amersham Pharmacia Biotech (Arlington Heights, IL). Ovine FSH and human CG were obtained from the National Hormone and Pituitary Distribution Program (Bethesda, MD). Pregnant mare's serum gonadotropin (PMSG) was a product from Teikokuzouki, Inc. (Tokyo, Japan). Diethylstilbestrol (DES) was purchased from Sigma (St. Louis, MO).

### Animals

Immature Wistar female rats (21 days old) were used. In order to obtain granulosa cells for primary culture, rats were treated with 2 mg DES in 0.1 ml of sesame oil once daily for 4 days to stimulate the proliferation of the cells. Cells were harvested, and primary culture was performed as reported previously [26]. For Northern blotting and in situ hybridization, rats were treated with either 30 IU of PMSG or with the same treatment for 48 h followed by the administration of 10 IU of hCG. At all times, the animals were treated according to National Institutes of Health guidelines.

### Subtraction cloning and DNA microarray

In order to identify the granulosa cell-differentiation related genes, subtraction cloning and DNA microarray analyses were performed. Rat granulosa cells in the primary culture under serum-free conditions are known to undergo cell differentiation from 12 to 24 h after FSH treatment. Therefore, cultured granulosa cells in 50 mm dishes containing 5.0 × 10^6 ^viable cells were treated with or without 30 ng/ml FSH for 15 h. Messenger RNAs from FSH treated cells as well as control cells were then extracted. They were converted to double stranded cDNAs, and subtraction cloning was performed as previously reported [27]. Resulting cDNAs of size-range between 0.5 – 2.0 kb long were isolated and cloned into a pGEM-T easy vector to construct a subtraction plasmid cDNA library. About 400 clones in the plasmid library were randomly picked up and their nucleotide sequences were partially determined.

For DNA microarray analysis, mRNAs from the cells treated with or without FSH were labeled with fluorescent dyes, Cy3 and Cy5, respectively. Labeling reaction were performed at 37°C for 1 h in the dark in a 30 microliter reaction mixture that contained 50 mM Tris-HCl (pH 8.3), 75 mM KCl, 3 mM MgCl_2_, 10 mM DTT, 2.5 mM Fluorolink Cy3- or Cy5-dCTP, dNTP mix (0.5 mM dATP, 0.5 mM dGTP, 0.5 mM dTTP and 0.2 mM dCTP), 600 units of Superscript II, 1.5 micrograms of mRNA and 1 microgram of oligo dT primer. Labeled cDNA probes were neutralized with 2 mM EDTA, 0.1 N NaOH and 0.1 N HCl and purified with Microcon YM-30 (Millipore, USA). The labeled cDNA pools were denatured by boiling at 95°C for 2 min with 4 × SSC, 25 × Denhartd's and 0.2 % SDS, and then mixed and hybridized to microarray glass slides containing 3200 cDNA elements (HyperGene Rat cDNA, DNA Chip Research Inc. Japan).

### Cloning and nucleotide sequence of full length cDNA for rat p120

In order to isolate full-length cDNA clones for rat p120, a cDNA library of rat granulosa cells was screened with a ^32^P-labeled 750 bp fragment of rat p120 (NM_001008509). Ten positive clones were isolated from 10,000 cDNA clones. Nucleotide sequence of one clone among them was determined from both ends by the dye terminator cycle sequencing method using an automated DNA sequencer.

### Plasmids

A human p120 (hp120/pKCR2) cDNA construct was kindly provided by Dr. Monden (Gunma University, Japan) [24]. A GRE-Luc reporter (cGREx2/pGL3-TK), human androgen receptor (hAR/pSG5), and a human progesterone receptor (hPR/pcDNA3) cDNA constructs were kindly provided by Dr. Kato (The University of Tokyo, Japan) [28]. An ERE-Luc reporter (ERE-TATA/pGL2) and estrogen receptor-alpha (rER-alpha/pSG5) cDNA constructs were kindly provided by Dr. Maruyama (Yamanouchi Pharm.Co.Ltd, Tokyo, Japan) [29]. A rat estrogen receptor-beta (rER-beta/pSV2) cDNA construct was kindly provided by Dr. Muramatsu (Research Center for Genomic Medicine, Saitama Medical School, Japan) [30]. All of the reporter plasmids were authenticated by DNA sequencing.

### Reverse transcription (RT) and polymerase chain reaction (PCR) of rat p120

Total RNA was isolated from various tissues using the TRIZOL reagent. Reverse transcription (RT) was performed at 37°C for 1 h in a 20 microliter reaction mixture that contained 50 mM Tris-HCl (pH 8.3), 75 mM KCl, 3 mM MgCl_2_, 0.5 mM dNTPs, 10 mM DTT, 200 units of Superscript II, 1 microgram of total RNA and 100 pmol of random hexamer. PCR was then performed using 50 microliter reaction mixture containing 2 mM Tris -HCl (pH8.0), 10 mM KCl, 2 mM MgCl_2_, 0.2 mM dNTPs, 10 pmol of PCR primers, 2 microliters of the reverse transcription product as a template, and 1.5 units of EXTaq DNA polymerase. The PCR was conducted at 94°C for 2 min, followed by 24 cycles 94°C for 20 sec, 54°C for 30 sec and 72°C for 45 sec. DNA fragments were amplified using a specific 5'- primer (CGCAAACTGACTGCTGAGAGAGTT, NM_001008509, 385–408) and a specific 3'- primer (CAGAGGTAGCCTCTTCCATAGTGG, NM_001008509, 710–733).

### Northern blotting

For Northern blot analysis, 10 micrograms of total RNA was separated by electrophoresis on a 1 % denaturing agarose gel, transferred to a nylon membrane (Biodyne, ICN Biomedicals, INC., Glen Cove, NY), and cross-linked by UV irradiation. Complementary DNA Fragments of p120, ER-alpha, ER-beta and PR, were radio-labeled using the *Bca *BEST DNA Labeling Kit, and the labeled products were used as the probe. The membrane was hybridized at 68°C for 1 h in the ExpressHyb hybridization solution with a ^32^P-labeled probe. The membrane was washed twice at 50°C for 40 min in 0.1 × SSC and 0.1 % SDS. The blot was also hybridized with a radio-labeled probe specific for rat glyceraldehyde 3-phosphate dehydrogenase (GAPDH) or acidic ribosomal phosphoprotein PO (36B4).

### Quantitative Real-time PCR

Total RNA was isolated from cultured granulosa cells using the TRIZOL reagent. Five micrograms of total RNA was reverse-transcribed and prepared single strand cDNA molecules. As an internal standard, TATA binding protein (TBP) was used. PCR reaction was performed using Power SYBR Green PCR Master Mix (Applied Biosystems). Serial dilutions of the templates were prepared and the relative standard curve method was used to quantitate mRNA levels for each target gene[31]. Each real-time RT-PCR was performed in a 25 microliter final volume containing 1.25 U/microliter of MultiScribe RT, 300 nM each forward and reverse primer. Specific primers used were; rat p120 (5'-GCCTGGAGGAGCCTAAGGA, 3'-TATGTATACTAAAGCCATCATCGCTTTC), ERβ (5'-TGCCCTGGTCTGGGTGAT, 3'-TGATGTGCCTGACGTGAGAAAG), PR (5'-TCAAGGCAATTGGCTTAAGACA, 3'-GAGCTGTTTCACAAGATCATGCA), and TBP (5'-TGCACAGGAGCCAAGAGTGAA, 3'-CACATCACAGCTCCCCACCA). Statistical analyses were carried out by analysis of variance. Differences between groups were evaluated with Duncan multiple comparison test. Data are expressed as mean ± SEM and P < 0.05 is considered significant.

### Transfection

CV-1 cells, originated from an African green monkey kidney cell, were maintained in Dullbecco's modified Eagle's medium supplemented with 10% fetal bovine serum and antibiotics in a humidified atmosphere containing 5% CO_2 _and 95% air at 37°C. Transfection was performed using the Lipofectamine PLUS reagent. All plasmids used for transfection were prepared using a QIAGEN plasmid kit, and purified by CsCl gradient ultracentrifugation. Cells (2 × 10^5^) were dispensed into 24-well plates and cultured for 24 h prior to transfection. DNA samples which contained each reporter plasmid and the pRL *Renilla *luciferase control vector (for normalization) with or without expression plasmids (hAR/pSG5, hPR-B/pcDNA3, rER-alpha/pSV2 or rER-beta/pSG5) were mixed and added to the cells in hormone-free conditions. Three hours after transfection, the medium was changed. Twenty-four hours later, the cells were treated with a ligand for each steroid hormone receptor (10 nM or 100 nM) for 24 h. Cells were harvested 48 h after the transfection. Measurements were made using a Lumat LB9501 luminometer (Berthold) in a single tube, with the first assay for firefly luciferase followed by the *Renilla *luciferase assay. Firefly luciferase activities (relative light units) were normalized to *Renilla *luciferase activities.

### In situ hybridization of rat p120

Rat ovaries were embedded into compounds and frozen in liquid nitrogen. Fourteen micrometer thick sections were cut by a cryostat and mounted on silane-coated glass slides for in situ hybridization. The sections were fixed in 4% paraformaldehyde in PBS for 15 min, washed with PBT (PBT: PBS plus 0.1% Tween-20), and acetylated in 0.25% acetic-anhydride in TEA (0.1 M Triethanolamine, 0.15 M NaCl pH8.0) for 10 min, and washed again with PBT. They were then dehydrated through ethanol step series (70–100%) and dried. Complementary DNA Frangments of rat p120 (AB180485, 385–733), ER-beta (NM012754, 49–584) and PR (NM022847, 2137–2697) were subcloned into the pGEM-T easy vector, respectively, and antisense or sense [^35^S] CTP-labeled RNA probes were synthesized using T7 or SP6 RNA polymerase. The samples were hybridized and washed at high stringency and autoradiographed with the emulsion of NTB2 (Eastman Kodak Co., Rochester, NY). All slides were counterstained with hematoxylin, dehydrated, and mounted.

## Results

**Figure 1:**
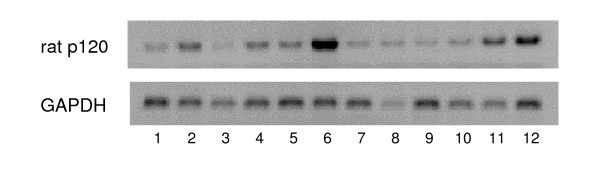
Tissue Distribution of rat p120 RT- PCR was performed using p120 specific primers. Total RNA was isolated from various tissues of adult rats. Ten micrograms of total, extracted RNA were reverse-transcribed, and a portion (1/100) was subjected to the PCR reaction for the specific amplification of p120 and GAPDH, respectively. The reaction mixtures were separated 1.5 % agarose gel and visualized with EtBr staining. The samples are brain (lane 1), pituitary (lane 2), liver (lane 3), adrenal (lane 4), uterus (lane 5), testis (lane 6), kidney (lane 7), spleen (lane 8), intestine (lane 9), stomach (lane 10), untreated ovary (lane 11), ovary treated with PMSG for 48 h (lane 12).

In order to isolate FSH inducible genes in cultured rat granulosa cells, a subtraction cloning and DNA microarray analyses were performed. Hundreds of candidate genes were picked-up and 10 genes among them were confirmed as FSH inducible by semi-quantitative RT- PCR analysis (Table. 1). As shown in Fig. 1, RT-PCR revealed that p120 was expressed ubiquitously, but strong expression of the mRNA was observed in the ovary (lane 11) and the testis (lane 6). PMSG treatment increased the p120 expression of mRNA after 48 h (lane 12), indicating that p120 was also induced in the ovary in vivo.

**Table 1:** Induced genes in the rat granulosa cells stimulated with FSH for 15 h.

**Accession**	**Definition**
AB180485	Human thyroid hormone receptor coactivating protein like protein (rat p120)
AB180912	Unknown serine protease like protein
U23776	Rat Eker rat-associated intracisternal-A particle element
NM_012600	Rat Malic enzyme 1, soluble (Me1)
AY009092	Rat retrovirus SC1
NM_011462	Mouse spindlin (Spin)
NM_019683	Mouse globin inducing factor, fetal (Gbif-pending)
M64393	Rat 3-alpha-hydroxysteroid dehydrogenase (3-alpha-HSD)
NM_022265	Rat programmed cell death 4 (Pdcd4)
NM_017290	Rat ATPase, Ca++ transporting, cardiac muscle, slow twitch 2 (Atp2a2)

**Figure 2:**
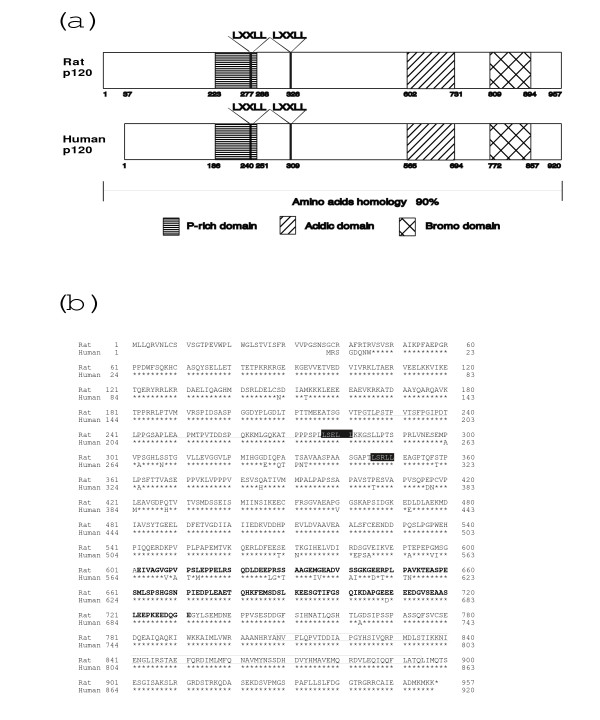
Structure of p120. (a), Schematic illustrations of the structures of rat and human p120. LXXLL motif (L, leucine; X, any amino acid). (b), A deduced amino acid sequence of rat p120. The amino acids are numbered starting from the initiation codon. The proline-rich domain between 223 and 288 amino acid residues from the N-terminus is underlined, and acidic amino acid-rich domain between 602 and 731 is shown by bold type. The bromo-domain between 809 and 894 is boxed. The putative NR box (LXXLL motif) is boxed by colored background.

We then cloned a full-length cDNA encoding p120 and the nucleotide sequence was determined. Fig. 2 shows the deduced amino acid sequence of rat p120, which is compared with that of human p120. It contains proline-rich, acidic, and bromo-domains, respectively, and there are two LXXLL-motifs between proline-rich and acidic domains. The sequences are well conserved between rat and human (94% identity at amino acid level), except that rat p120 has 45 amino acid residues extended at N-terminus.

**Figure 3:**
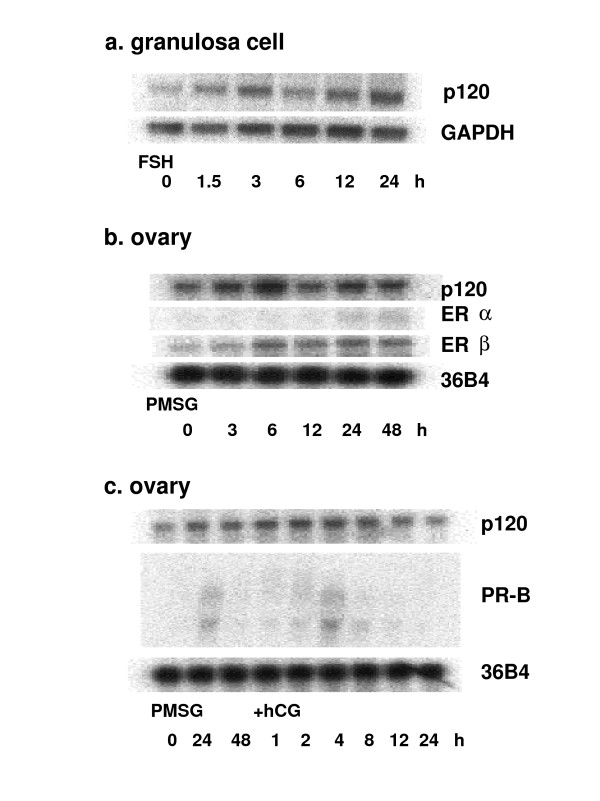
Induction of rat p120 gene expression in the ovary. Northern blot analysis of rat p120 mRNA in cultured granulosa cells treated with FSH (a), in immature rat ovary primed with PMSG (b), and in immature rat ovary primed with PMSG followed by hCG (c). Total RNA was isolated at the indicated times after the treatments. All samples contained 10 micrograms of total RNA. The blots were hybridized with each specific probe of rat p120, ER-alpha, ER-beta, or PR, respectively. The blots were striped and rehybridized with a probe specific for GAPDH or 36B4 to normalize for equivalent loading of RNA.

**Figure 4:**
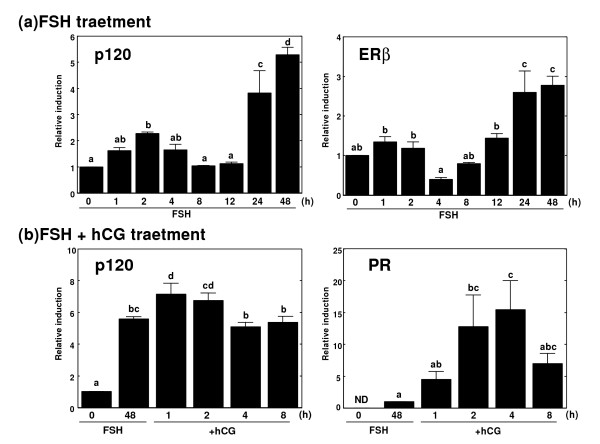
Induction of rat p120 gene expression in the ovary. Real-time PCR was performed using each specific primer of rat p120, ER-beta, or PR, respectively. Total RNA was isolated from cultured granulosa cells treated with FSH for 48 hr (a) or with FSH for 48 h followed by hCG (b). Each value represents the mean ± SEM of 4 independent Real-time PCR experiments. Different letters for the same graph indicate statistically significant differences (P < 0.05; Duncan multiple comparison test).

Gene expression of p120 in the cultured rat granulosa cells was analyzed by Northern blotting (Fig. 3) and real-time RT-PCR (Fig. 4). Rat granulosa cells cultured under serum free conditions were treated with 30 ng/ml of FSH. Gene expression of p120 was rapidly induced by the FSH treatment within 1 h, with a transient peak at 2 h, and increased again 24 h after the treatment. The treatment of immature rats with PMSG in vivo also induced the gene expression of p120 in the ovary with a transient peak at 6 h after the treatment, and then the gene expression was increased again 24 h after the treatment. Gene expression level of estrogen receptor-alpha (ER-alpha) in the ovary of immature rat was very low even after the PMSG treatment. On the other hand, ER-beta was induced in the ovary by the PMSG treatment. The hCG administration 48 h after the PMSG treatment further increased the p120 gene expression. Levels of progesterone receptor (PR), which is known as one of the ovulation related genes, were also shown. These two genes, p120 and PR, showed similar mRNA expression patterns.

**Figure 5:**
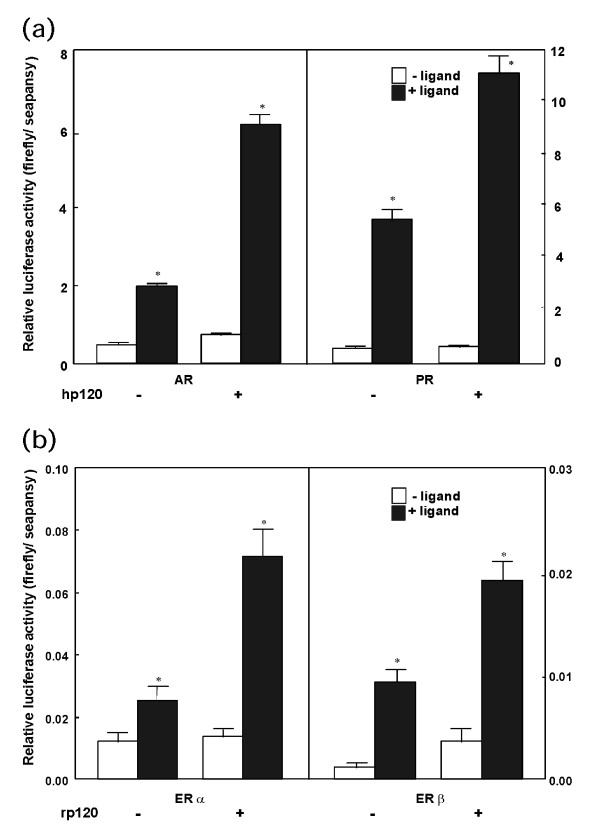
Activation of nuclear hormone receptors with p120. CV-1 cells were transfected as described in Materials and Methods with cGREx2/pGL3-TK and hAR (hAR/pSG5), or with cGREx2/pGL3-TK and hPR (hPR/pcDNA3) in the presence or absence of human p120 (a), ERE-TATA/pGL2 and rER-alpha (rER-alpha/pSV2), or ERE-TATA/pGL2 and rER-beta (rER-beta/pSG5) in the presence or absence of rat p120 (b). Ligands for each nuclear receptor (10^-8^M testosterone propionate, 10^-8^M progesterone, 10^-8^M estradiol, and 10^-7^M estradiol) were also added to the cultures. Each value represents the mean ± SEM of 4 independent transfection experiments. *P < 0.01 by Student's *t *test.

In order to verify the co-activator function of rat and human p120, co-transfection experiments were done using various steroid hormone receptor genes and reporter genes. CV-1 cells were co-transfected with a luciferase reporter vector and steroid hormone receptor cDNA expression vectors in the presence or absence of their ligand steroid hormones. As shown in Fig. 5a, luciferase activities were increased by the addition of either testosterone propionate or progesterone when AR or PR expression vectors were co-transfected to the cells, respectively. Co-transfection of a human p120 expression vector further increased the luciferase activities in the presence of the ligands. Similarly, co-transfection of a rat p120 expression vector with either ER-alpha or ER-beta also increased the luciferase activities in the presence of estradiol as shown in Fig. 5b. These observations clearly indicate that p120 works as a transcriptional co-activator of androgen, progesterone and estrogen receptors.

**Figure 6:**
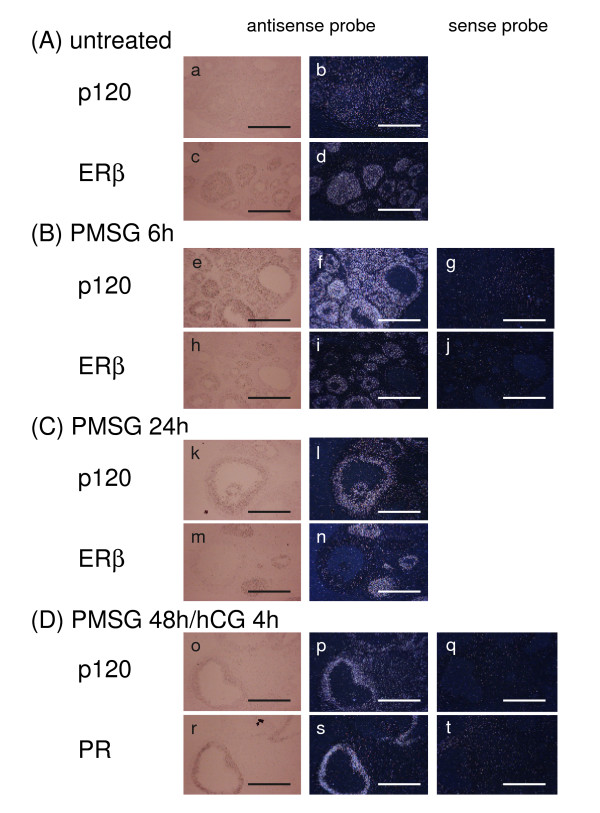
In situ hybridization of p120 mRNA in the immature rat ovary. Ovaries from 21-day-old immature rats were dissected, sectioned and hybridized with ^35^S-labeled cRNA probes. All sections were shown by both dark-field illumination and bright-field photomicrographs of hematoxylin-staining. (A) Sections from untreated ovaries hybridized with p120 (a, b) or ER-beta (c, d) antisense probes, (B) PMSG treatment for 6 h hybridized with p120 (e, f) or ER-beta (h, i) antisense probes. Sections were also hybridized with sense probes (g, j), (C) PMSG treatment for 24 h hybridized with p120 (k, l) or ER-beta (m, n), (D) hCG treatment for 4 h after PMSG treatment hybridized with p120 (o, p) or PR (r, s) or sense probes (q, t). Original magnification was × 100. *Scale bars*, 0.1 mm.

Localization of rat p120, ER-beta and PR in the ovary was examined by in situ hybridization using adjacent frozen sections as shown in Fig. 6A–D. Immature female rats were treated either with PMSG or PMSG/hCG. Gene expression of p120 was weakly detected throughout the ovary of untreated rats (a, b), whereas ER-beta was expressed predominantly in the granulosa cells of small preantral and antral follicles of untreated rats (c, d). Strong gene expression of p120 both in granulosa and theca cells was observed 6 h after the PMSG treatment (e, f), whereas ER-beta was expressed mainly in the granulosa cells of small and midsize follicles (h, i), indicating that p120 and ER-beta are partly co-localized in the ovary 6 h after the PMSG stimulation. Gene expression of p120 was also observed in the stroma. However 24 h after the PMSG treatment, p120 was strongly expressed in the granulosa cells of large antral follicles where practically no expression of ER-beta mRNA was observed as shown in Fig. 6C (k-n). In addition, a level of p120 was gradually decreased 48 h after the PMSG treatment (data not shown), but by the following treatment with hCG, strong expression of p120 mRNA was re-induced in the granulosa cells of large antral follicles 4 h after the treatment (o, p). The hCG treatment was a typical protocol for the induction of ovulation from large antral follicles. This was confirmed by the fact that expression of PR mRNA, that is known to be induced in the ovulatory follicles just before the ovulation, was induced by the hCG treatment in the granulosa cells of the same large antral follicles that expressed p120 (r, s).

## Discussion

As shown in Table 1, a rat homologue of co-activator, p120, was identified as one of the gonadotropin inducible genes in the ovarian granulosa cells. The co-activator p120 has been originally identified as a protein that interacts with thyroid hormone receptor (TR) -beta1 at the ligand-binding domain (LBD) [24]. Monden *et al*. reported that human p120 consists of 920 amino acid residues with two LXXLL motifs and a bromo-domain, and interacts with TR-beta1 and PPAR-gamma to increase their transcriptional activities [25]. The LXXLL-motifs are found in most co-activators such as RIP140, SRC-1, TIF-2 and CBP/p300, and is shown to interact with common AF-2 regions of nuclear hormone receptors [19,20,32]. The bromo-domain motifs are also often found in co-activators, and are reported to be essential for exhibiting the full activity of p300 in terms of histone acetyltransferase (HAT) activity [33,34]. The bromo-domain motifs of Gcn5p, PCAF, TAF250 and p300 are reported to interact directly with histone molecules [35-37]. In this study, we examined ability of p120 to activate various steroid hormone receptors. As shown in Fig. 5, p120 activated all the steroid hormone receptors examined in the presence of their ligand steroid hormones. This is consistent with the structural information described above. Therefore, together with the previous findings, p120 acts as a co-activator of most steroid hormone receptors.

Co-activators SRC-1 and CBP/p300 are reported to be ubiquitously expressed, whereas TIF2 and Rip140 show some tissue specific expression [33,38,39]. Expression of rat p120 mRNA was observed in most tissues but was highest in gonads, suggesting some roles for gonadal functions. Knockout mice of steroid receptor co-activator 3 (SRC-3), a member of p160 family, exhibit infertility [22], and those of nuclear receptor interacting protein 1 (Nrip1/RIP140) are also infertile due to the blockage of the oocytes release from the Graafian follicles by the ovulatory stimuli [23]. These reports indicate that co-activators are also important for the follicular development and ovulation.

With respect to the function of steroid hormone receptors in the ovary, ER-beta and PR were expressed in the ovary in a gonadotropin dependent manner as demonstrated by Northern blotting and in situ hybridization. However, induction patterns of ER-beta and PR are quite different. Even after the PMSG stimulation, ER-beta gene expression was mainly observed in granulosa cells of pre-antral or small antral follicles, whereas expression of PR mRNA was found in the granulosa cells of large antral follicles after the stimulation. In situ hybridization revealed that the induction pattern of p120 was very similar to that of PR. It is well established that PR participates in the ovulation process occurring in the pre-ovulatory large antral follicles. Ovulation is triggered by LH surge from the pituitary. About 4 h after the LH surge, expression of PR mRNA is rapidly induced in the granulosa cells of pre-ovulatory large antral follicles destined to ovulate [7,8]. Progesterone binds to the induced receptor to activate gene expression of specific proteases, ADAMTS-1 or Cathepsin L, which are thought to work on follicle rupture by degrading the ovulating follicle walls [40]. In addition, female PR knockout mice are infertile due to the blockage of ovulation [12,41]. In the present experiments, we showed that the induction and tissue distribution patterns of p120 and PR were very similar, and that p120 was able to enhance the transcriptional activity of PR in the presence of progesterone. It is likely that p120 may also be one of the co-activators that participate in the ovulation from pre-ovulatory follicles.

## Conclusion

Rat p120 was strongly expressed in the granulosa cells of pre-ovulatory follicles by the stimulation with gonadotropins. Rat p120 was able to enhance the transcriptional activity of progesterone receptor in the presence of progesterone, and p120 co-localized with progesterone receptor in the pre-ovulatory follicles. These observations suggest that p120 may work together with PR in the granulosa cells of ovulatory follicles to promote the ovulatory process in the ovary.

## Authors' contributions

MY participated in the histochemical studies, transfection of genes and moleculer analysis of genes. TM, KY, TY, HO, TS, and TK participated in the moleculer analysis of genes. KM participated in the design of the study.
